# Pericardial and Pleural Effusions in Non-ICU Hospitalized Patients with COVID-19—A Retrospective Single-Center Study

**DOI:** 10.3390/jcm13133749

**Published:** 2024-06-27

**Authors:** David V. Mangaloiu, Cătălin Tilișcan, Alexandra D. Răriș, Anca R. Negru, Violeta Molagic, Constanta A. Vișan, Laurențiu M. Stratan, Nicoleta Mihai, Ștefan S. Aramă, Victoria Aramă

**Affiliations:** 1 Faculty of Medicine, “Carol Davila” University of Medicine and Pharmacy, 37 Dionisie Lupu Street, 020021 Bucharest, Romania; david-valentin.mangaloiu@drd.umfcd.ro (D.V.M.); anca1808@yahoo.com (A.R.N.); violeta_molagic@yahoo.com (V.M.); angelica.visan@yahoo.com (C.A.V.); laurentiustratan@gmail.com (L.M.S.); nicoleta.iftode@yahoo.com (N.M.); sorinaarama@gmailc.om (Ș.S.A.); dr.arama@mateibals.ro (V.A.); 2 National Institute for Infectious Diseases “Prof. Dr. Matei Balş”, 021105 Bucharest, Romania; 3 Emergency Clinical Hospital for Children Marie Curie, 041451 Bucharest, Romania; alexandra.raris@gmail.com

**Keywords:** COVID-19, pericardial and pleural effusions, risk factors, hospitalized

## Abstract

**Background**: Pericardial and pleural effusions are two complications recently described in patients hospitalized with COVID-19 infections. There are several mechanisms that have been proposed and refer to SARS-CoV-2’s capacity to bind to cell surfaces via various receptors and its broad tissue tropism that might cause significant complications. The aim of the present study is to evaluate the incidence of pericardial and pleural effusions during COVID-19 infection as well as to determine the risk factors associated with these complications. **Methods**: We conducted a retrospective single-center study that included 346 patients admitted to the National Institute of Infectious Disease “Prof. Dr. Matei Bals” (Bucharest, Romania), from 1 January to 25 May 2021, during the third wave of the pandemic. Socio-demographic and anthropometric data were collected for each patient. The patients were evaluated clinically, biologically, and radiologically within 48 h of admission. Patients were divided into 3 groups: (1) patients with pericardial effusions—18; (2) patients with pleural effusions—28; (3) patients without pericardial/pleural effusions—294. **Results**: After exclusion criteria were applied, 337 patients were analyzed. The median age of the participants was 58.26 ± 14.58 years. More than half of the hospitalized patients had associated respiratory failure (61.5%), of which 2.7% had a critical form of the disease and 58.8% had a severe form. The cumulative percentage for pericardial and pleural effusions for the study group was 12.8% (43 patients out of 337). The prevalence of pericardial effusion was 5.3%, twice more frequent among male respondents. Pleural effusion was identified in 8.3% patients. Most patients had unilateral effusion (17), compared to 11 patients who had bilateral involvement. Based on laboratory results, patients with pericardial and pleural effusions exhibited increased levels of C reactive protein, erythrocyte sedimentation rate, NT proBNP, and a higher value of neutrophil/lymphocyte count ratio. In contrast to patients without pleural and pericardial effusions, those with these symptoms experienced a higher frequency of severe or critical illness and longer hospital stays. **Conclusions**: Pericardial and pleural effusions can complicate COVID-19 infections. In our study, the prevalence of pericardial and pleural effusions in hospitalized patients was low, being associated with the same comorbidities and a number of clinical and biological parameters.

## 1. Introduction

COVID-19 is a highly contagious viral infection that has been responsible for important mortality and morbidity during the last 4 years since the pandemic started. With more than 775 million confirmed cases and 7 million deaths attributable to SARS-CoV-2—Severe Acute Respiratory Syndrome Coronavirus 2—the COVID-19 pandemic has presented a formidable challenge to global public health, having at the same time an indelible mark on economic and social level [1,2].

SARS-CoV-2 (a single-stranded positive RNA-enveloped virus that belongs to the Beta-coronavirus genus) is responsible for COVID-19, which primarily targets the respiratory tract, gaining entry through angiotensin-converting enzyme 2 (ACE2) receptors present on respiratory epithelial cells [3,4]. While respiratory symptoms dominate the clinical presentation of COVID-19, emerging evidence suggests that the virus’s impact extends beyond the pulmonary tract, involving multiple organ systems and leading to an increased number of extrapulmonary manifestations such as neurological, cardiovascular, hepatobiliary, renal, gastrointestinal, dermatological and endocrinological difficulties [4,5,6]. Among these manifestations, pericardial and pleural effusions have emerged as notable entities, raising questions about their pathogenesis and clinical significance [7].

Characterized by abnormal accumulation of fluid within the pericardial sac and pleural cavity, pericardial and pleural effusions are not exclusive to COVID-19, being found in other diseases [8]. Viral infections, such as those caused by the influenza virus, coxsackievirus, cytomegalovirus, and Epstein-Barr virus, as well as bacterial pathogens, including Streptococcus pneumoniae, Staphylococcus aureus, Mycobacterium tuberculosis, and Haemophilus influenzae, can induce pericardial and pleural effusions. Therefore, understanding the pathophysiological mechanisms driving pericardial and pleural effusions in the context of SARS-CoV-2 infection is crucial for elucidating the disease’s full spectrum and guiding therapeutic interventions [9,10,11].

Several potential mechanisms have been proposed to explain these phenomena. Firstly, direct viral invasion of the serosal membranes might induce local inflammation and increased vascular permeability, resulting in fluid leakage. SARS-CoV-2 binds to the angiotensin-converting enzyme 2 (ACE2) receptors, which are expressed in various tissues, including the pericardium and pleura, facilitating viral entry and subsequent inflammatory response [4,12].

Secondly, the systemic inflammatory response syndrome (SIRS) induced by COVID-19 can lead to a cytokine storm, characterized by the release of large quantities of pro-inflammatory cytokines such as interleukin-6 (IL-6), interleukin-1β (IL-1β), and tumor necrosis factor-alpha (TNF-α). This cytokine storm can increase vascular permeability and capillary leakage, promoting the formation of effusions. Additionally, COVID-19-associated coagulopathy, with elevated levels of D-dimer and other markers of thrombosis, can contribute to effusion formation through microvascular thrombosis and impaired lymphatic drainage [13,14,15].

Moreover, pre-existing conditions and comorbidities in COVID-19 patients, such as heart failure, chronic kidney disease, and liver cirrhosis, can exacerbate the tendency to develop effusions. These underlying conditions may lead to fluid overload or impaired resorption of serous fluids, thereby compounding the effects of COVID-19 on the serosal cavities [16,17]. 

Pericardial and pleural effusions in the context of COVID-19 can present with a wide spectrum of clinical manifestations, ranging from asymptomatic incidental findings to severe, life-threatening complications such as cardiac tamponade and respiratory compromise [18]. Reports from diverse clinical settings have documented the occurrence of these findings in COVID-19 patients, either as isolated findings or as part of a constellation of extrapulmonary manifestations. Several studies have investigated the prevalence and clinical significance of pericardial and pleural effusions in patients with COVID-19. Early observations and case reports indicated that these effusions were relatively uncommon but could occur in severe cases. Subsequent larger studies and reviews have provided more detailed insights. For instance, a study by Al-Tarbsheh et al. (2022) using echocardiographic data from 195 patients with COVID-19 found that pericardial effusion was present in approximately 4.6% of cases, often associated with higher disease severity and poorer outcomes [8]. Similarly, Lazar et al. highlighted its prognostic implications after analyzing 100 patients with severe COVID-19, finding a prevalence of 27% for pericardial effusion. Furthermore, patients with pericardial effusions showed elevated levels of cardiac enzymes (myoglobin, CK, and CK-MB), LDH, platelets, and CRP, along with a higher mortality rate [19]. Regarding pleural effusion, a retrospective analysis by Cappeli et al. (2023) reported a notable prevalence of 23% in patients hospitalized with COVID-19. The study concluded that pleural effusion serves as a negative prognostic factor, significantly associated with increased disease severity, extended hospital stays, greater utilization of healthcare resources, and higher mortality rates [20].

The clinical implications, prevalence, outcomes, and associated risk factors of these effusions are still being elucidated. In this context, and considering the numerous unanswered questions regarding these complications, the current study aims to evaluate the incidence of pericardial and pleural effusions in patients hospitalized with COVID-19, as well as to identify the risk factors associated with these conditions.

## 2. Materials and Methods

### 2.1. Study Design and Participants 

We conducted a retrospective cohort study which included 346 patients admitted to the National Institute of Infectious Disease “Prof. Dr. Matei Bals” (Bucharest, Romania). The patients included were hospitalized in a non-intensive care unit (non-ICU), from 1 January to 25 May 2021, during the third wave of the pandemic. 

The study inclusion criteria were represented by patients aged over 18 years with a confirmed COVID-19 diagnosis via a Real Time-Polymerase Chain Reaction test (RT-PCR). We excluded from the studied group patients aged <18 years, pregnant women due to the impossibility of being evaluated by computer tomography, and patients without computer tomography or only with lung radiography on admission. After applying these criteria, 9 patients were excluded. Patients were divided into 3 groups: (1) patients with pericardial effusions—18, (2) patients with pleural effusions—28, and (3) patients without pericardial/pleural effusions—294. In total, the study group was formed by 337 patients. Three patients developed both pericardial and pleural effusions. 

To confirm the diagnosis, all the patients were tested by collecting oropharyngeal and nasopharyngeal swab specimens, which were analyzed through RT-PCR. All the patients tested positive for SARS-CoV-2. 

Regarding the COVID-19 disease severity staging, according to WHO (World Health Organization), patients were divided into a critical form—the presence of ARDS, sepsis or septic shock, or any other condition that would typically call for the administration of life-sustaining treatments like mechanical ventilation (invasive or non-invasive) or vasopressor therapy; a severe form—severe pneumonia, oxygen saturation on room air < 90–94%, and signs of severe respiratory distress (respiratory rate > 30/min, accessory respiratory muscle use); and a non-severe form—the absence of criteria for critical or severe COVID-19 [21]. None of the patients included in the study required pericardiocentesis or thoracentesis due to the small quantity of pericardial and pleural effusions.

### 2.2. Study Variables

We collected demographic and anthropometric information as well as clinical and paraclinical data. Demographic and anthropometric data were represented by age, gender, weight, height, and body mass index (BMI). Concerning clinical and paraclinical parameters, we included the following parameters: symptoms at admission, comorbidities, respiratory function parameters (pulse-oximetry, and/or respiratory rate), complete blood count, neutrophil/lymphocyte count ratio, coagulation, biochemical parameters (lactate dehydrogenase (LDH), N-terminal pro-B-type natriuretic peptide (NT-proBNP), troponin I (TnI), myoglobin, hemoglobin A1c (HbA1c) and inflammatory markers (C-reactive protein (CRP), erythrocyte sedimentation rate (ESR), fibrinogen, interleukin 1 (IL 1), interleukin 6 (IL 6), serum ferritin, and tumor necrosis factor (TNF alfa).

### 2.3. Ethical Considerations

The Ethics Committee of the National Institute of Infectious Diseases “Prof. Dr. Matei Bals” approved the study (C14730/16.12.2021), and data collection was conducted in alignment with the Helsinki Declaration and national regulations. Given the retrospective nature of the study, informed consent was waived, as the study solely relies on anonymized and de-identified patient data. Patient privacy was rigorously protected throughout the study.

### 2.4. Statistical Analysis

Statistical analysis was performed with SPPS 26 (Statistical Package for Social Science). Patient data are presented for quantitative variables as mean, median, standard deviation, and percentage for categorical variables. The Chi-square test was utilized for categorical data and the Mann–Whitney test for continuous variables. Univariable statistical analysis was performed using an independent *t*-test and a Pearson’s chi-square test. We used Fisher’s exact test in the analysis of contingency tables for categorical variables. All tests of significance were two-tailed and a *p*-value < 0.05 was considered significant. 

## 3. Results

### 3.1. Socio-Demographic Aspects and Patient Characteristics 

We enrolled 337 patients confirmed with a COVID-19 infection in our study. The median age of the participants was 58.26 ± 14.58 years. Approximately two-thirds of the patients were men, the female/male ratio being 1.55/2.8. More than half of the hospitalized patients had associated respiratory failure (61.5%), of which 2.7% had a critical form of the disease and 58.8% had a severe form. The median value of the body mass index for the studied group was 28.71 kg/m^2^. At admission, 98.5% of patients were symptomatic, the most frequent symptoms being cough (83%), fever (73.9%), asthenia (63.2%), myalgia (48.5%), chills (47.4%), headache (37.2%), arthralgia (25.9%), diarrhea (24.9%), anosmia (12.6%), and ageusia (9.4%).

The most frequent comorbidities reported in the studied group were arterial hypertension (49%), diabetes mellitus (17.85%), chronic viral hepatitis (6.2%), chronic renal disease (5.6%), and chronic obstructive pulmonary disease (2.1%). Approximately 41% of patients included were without any comorbidities. The percentages of comorbidities were slightly higher in patients with pleural and pericardial effusions than those without. These differences are statistically significant for hypertension and chronic renal disease for both groups and for chronic viral hepatitis only for Group 1 (Table 1).

The median length of hospitalization for all patients was 10.64 ± 5.36 days, with a minimum of 2 days and a maximum of 46 days. Patients with pericardial and pleural effusions required a longer period of hospitalization, respectively, 11.24 days and 11.96 days. The mean period of hospitalization for individuals with pericardial and pleural effusions was 11.67 days, compared with those without, which had a mean hospitalization of 10.50 days (*p* = 0.172). 

The mortality rate for the studied group was 1.2%—4 patients, of which 3 deaths were in the group without complications and 1 death in the group with pleural effusion. 

Table 1 presents socio-demographic data, comorbidities, disease severity, and the median number of days from symptoms onset to hospital admission for the studied group.

### 3.2. Prevalence of Pericardial and Pleural Effusions

The cumulative percentage for pericardial and pleural effusions in the study group was 12.8% (43 patients out of 337). The prevalence of pericardial effusion was 5.3% and was twice as frequent among male respondents. The minimum value was 3 mm, while the maximum value was 13 mm. The median value was 6.50 mm. In only 4 patients was the pericardial effusion identified in the first 7 days of hospitalization. For the others, it was identified at hospital admission. 

Pleural effusion was identified in 28 patients. The prevalence of this complication was 8.3%. Most patients had unilateral effusion (17), compared to 11 patients who had bilateral involvement. The minimum value was 2 mm, while the maximum value was 88 mm. We observed that patients with pleural involvement had a longer hospitalization than those without pericardial/pleural effusions (11.96 days vs. 10.50 days). However, it was not statistically significant. 

### 3.3. Risk Factors of COVID-19-Related Pericardial and Pleural Effusions

In the pericardial effusion group, we observed a higher age compared with the group without pericardial effusion (66.89 vs. 57.52 years), with a *p*-value showing a statistical correlation (*p* = 0.010). This indicates that, in our study, older age is significantly associated with the development of pericardial effusion. Regarding sex distribution, no significant differences were observed between the two groups. Although the body mass index (BMI) was lower in this group (26.39 ± 3.14), it appears that a higher BMI is correlated with the development of pericardial effusion (*p* = 0.024). This suggests that despite the lower average BMI, individuals with higher BMI are more prone to pericardial effusion.

Examining the profile of patients with pericardial effusions, we found that all patients developed symptoms, with fever and chest pain being the most frequent. Additionally, arterial hypertension, chronic renal failure, and chronic hepatitis were more common in this group. At least one comorbidity was present in 61.1% of cases with a pericardial effusion, indicating a higher burden of underlying health conditions in these patients. Considering laboratory parameters, there was a significant statistical correlation with the following:

- Neutrophil/lymphocyte count ratio (*p* = 0.029, 95% CI, −5.44:−0.29), suggesting that an elevated ratio is associated with pericardial effusion. In our study, the median value for this group was 9.15 ± 8.45.
- Elevated C-reactive protein levels (*p* = 0.050, 95% CI, −63.47:−0.02) were also correlated with pericardial effusion, highlighting the role of systemic inflammation. The median value was 101.82 ± 76.00.
- N-terminal pro-B-type natriuretic peptide (NT-proBNP) levels were significantly correlated (*p* = 0.000, 95% CI, −3451.80:−1178.50), indicating cardiac stress or dysfunction as a contributing factor. The median value was 2723.44 ± 5364.34.

Patients with pericardial effusions had a longer period from symptom onset to admission compared to those without (9.33 vs. 8.56 days), suggesting that delays in seeking medical care may contribute to the development or worsening of pericardial effusion.

The development of pleural effusion was correlated with arterial hypertension (*p* = 0.011), chronic renal failure (*p* = 0.001), and the number of days from symptom onset to admission (*p* = 0.009). These correlations suggest that patients with arterial hypertension and chronic renal failure are more prone to fluid accumulation in the pleural cavity, and a delay in hospital admission after the onset of symptoms. 

Regarding clinical and biological parameters, we found a significant statistical correlation between pleural effusion and the following parameters at the time of admission:

- Dyspnea (*p* = 0.028, 95% CI, −0.40:−0.20). Patients who presented with dyspnea at admission were more likely to develop pleural effusion. Dyspnea, as a symptom of respiratory distress, may indicate a greater severity of the disease, contributing to fluid accumulation in the pleural space.
- Severe form of the disease (*p* = 0.014, 95% CI, −0.38:−0.4). Pleural effusion was more common in patients with severe forms of the disease, of which 78% developed this complication.
- Acute respiratory failure (*p* = 0.001, 95% CI, −0.40:−0.11). The development of acute respiratory failure was strongly linked to the presence of pleural effusion, highlighting that fluid accumulation in the pleural cavity can contribute to the deterioration of respiratory function. Patients with pleural effusion had a respiratory rate of 20.29 ± 4.54 per minute and a blood oxygen saturation of 89% ± 54%.
- Lymphocyte count (*p* = 0.000, 95% CI, 234.37:481.32). There was a significant correlation between the decrease in lymphocyte count and pleural effusion. A lower number in the lymphocyte count may reflect a pronounced inflammatory response, which can be associated with pleural fluid accumulation. The median value was 702.14 ± 281.36.
- Neutrophil/lymphocyte count ratio (*p* = 0.013, 95% CI, −8.00:−1.02): A higher neutrophil/lymphocyte ratio was associated with pleural effusion, suggesting a disproportionate inflammatory response that may contribute to this complication. In our study, the median value of NLR was 10.58 ± 8.90.
- Erythrocyte sedimentation rate (*p* = 0.043, 95% CI, −26.88:−0.43). A higher value was correlated with pleural effusion. Patients in this group had a median value of 56.61 ± 26.76.
- C-reactive protein (*p* = 0.045, 95% CI, −66.85:−0.77): Elevated levels of C-reactive protein, a marker of acute inflammation, were significantly associated with pleural effusion in our study, highlighting the link between intense inflammation and fluid accumulation in the pleural cavity. The median value was 102.83 ± 80.07, higher than the value found in the other groups (Table 2).

We performed a multivariate binary logistic regression model, including the presence of pleural and pericardial effusions as dependent variables, and independent variables that had a significant statistical correlation in the univariate analysis (neutrophils, lymphocytes count, Ne/Ly, CRP, PAI-1, and NT proBNP. For pleural effusion, neutrophils, lymphocyte count, and PAI-1 remained significantly associated (Nagelkerke R Square = 0.23, *p* = 0.00). For pericardial effusions, only NT pro-BNP showed a significant correlation in the multivariate analysis (Nagelkerke R Square= 0.15, *p* = 0.05).

Table 2 presents the clinical, biological, and radiological characteristics of patients with and without pericardial and pleural effusions for the studied group. 

## 4. Discussion

Pericardial and pleural effusions can occur at a relatively low rate during COVID-19 infection, with reported incidences in most studies of approximately 5% and 8%, respectively, although these complications have been also documented by different authors with prevalence ranging widely from 4.6% to 90.7% for pericardial effusion and from 1% to 62.5% for pleural effusion, particularly among critically ill patients [8,22,23]. 

Parapneumonic effusion can complicate all cases of pneumonia. Among hospital-sized patients, the occurrence of parapneumonic effusion varies between 10% and 21%. This complication is more commonly observed in bacterial pneumonia than in viral pneumonia [24]. Pleural abnormalities in COVID-19 were initially thought to be either less frequent or were not fully recognized in the first months of the pandemic. Early in the disease, localized pleural thickening near the affected lung tissue and pleural retraction may be observed. Serious complications like pneumothorax, which can be life-threatening, are rare [25].

The invasion of the pulmonary tissue by SARS-CoV-2 triggers intense inflammation, leading to widespread damage to the alveoli and endothelial cells by inflammatory agents. Consequently, there is an escalation in interstitial fluid due to increased permeability of the microvasculature. This excess fluid migrates to the pleural spaces through the visceral pleura, driven by the interstitial-pleural pressure gradient. Moreover, direct viral invasion, inflammation of the visceral pleura, and the presence of inflammatory cytokines further enhance the permeability of the pleural surfaces. Postmortem examinations have detected positive SARS-CoV-2 PCR in pleural fluid, indicating direct viral invasion into the pleural space and the potential risk of transmission during pleural fluid handling [26].

When examining the incidence of pleural effusion associated with COVID-19, there is a notable emphasis on observational studies and reviews compared to pericardial effusion. In our study, the pleural effusion was unilateral in most patients, similar to other reports. In a systematic review and meta-analysis, Rathore SS et al. reported a prevalence of 9.55% of pleural effusion. They also found that pleural effusion was associated with increased severity of disease and mortality due to illness, compared with those without pleural effusion. The authors also reported that the presence of pleural effusion was correlated with increased disease severity and mortality rates compared to patients without this complication [27]. Regarding mortality risk, a retrospective observational study by Cappelli S et al. involving 681 subjects, including 166 patients with pleural effusion and 515 without, identified pleural involvement as an independent predictor of mortality. We do not confirm this association in our study, probably due to the lower number of enrolled subjects. Patients with this complication were more likely to require mechanical ventilation [20].

In our study, the prevalence of pleural effusion was consistent with those previously reported, at 8.3%. Patients presenting with this complication experienced a prolonged hospitalization period, with an average duration of 11.96 days compared to 10.50 days for those without pleural effusion. Also, in our study, other risk factors were associated with the development of pleural effusion, including lymphopenia and elevated levels of the neutrophil-to-lymphocyte count ratio, erythrocyte sedimentation rate, N-terminal pro-B-type natriuretic peptide (NT-proBNP), troponin, and C-reactive protein. As expected, symptoms such as fever, cough, dysphagia, and asthenia were more frequently observed in COVID-19 patients with pleural effusion compared to those without. Additionally, respiratory failure was significantly associated with pleural effusion, with most patients in the pleural effusion group exhibiting this complication.

Patients with pleural effusion also had a greater requirement for oxygen therapy compared to those without, with a median oxygen volume of 9.54 L per minute versus 7.62 L per minute. The mortality rate in the pleural effusion group was recorded at 3.6%.

Pericardial involvement can occur secondary to several viral infections, including EBV and CMV, as well as coxsackie viruses A and B, echovirus, adenoviruses, parvovirus B19, HIV, and influenza [10]. Regarding SARS-CoV-2, the pathophysiology of pericardial effusion is not yet fully elucidated, but there are several hypothesized mechanisms. First, the direct involvement of the pericardium, because of SARS-CoV-2 attachment via angiotensin-converting enzyme 2 (ACE-2) receptor, which is found in a wide range of cells, including cardiac fibroblasts, vascular endothelium, and cardiomyocytes. Secondary to SARS-CoV2 binding, the ACE-2 pathway is activated and generates myocardial injury and cardiomyopathy, which might be responsible for pericardial effusion [4,12]. Although this mechanism is still unclear, there are studies that report the isolation of SARS-CoV-2 in the pericardial fluid [28]. The indirect effects of the virus, secondary to oxidative stress-induced and cytokine storm, have been suggested as another responsible mechanism. Another pathological pathway is represented by the dysregulation of the immune system and, because of the activation of endothelial cells and macrophages, a hyperinflammatory phase appears during COVID-19 with the enormous release of TNF-α, IL-1, IL-2, IL-6, IL-8, and chemokines. This might be responsible for pericardial and myocardial involvement, as already some studies have shown the presence of interstitial mononuclear cell infiltration in the myocardium after autopsies were performed [13,14,15]. 

The prevalence of pericardial effusion in our study (5.3%) is similar to the one reported in the literature. In a retrospective study, Al-Tarbsheh AH et al. found a similar prevalence of 4.6% [8]. Pericardiac effusion with or without signs of pericarditis was reported mostly during the acute COVID-19 phase, although there is an increasing number of authors describing pericardial involvement as a delayed complication of SARS-CoV-2 infection, probably caused by the release of proinflammatory cytokines (interleukin [IL]-1β, IL-6, IL-8, IL-2 and tumor necrosis factor-α) [4,29]. 

No deaths were recorded in our study in the pericardial effusion group. Even if some studies are suggesting that pericardial effusion is a result of a severe inflammatory process and cytokine storm, as described above, in the studied group, we found a significant statistical correlation only with CRP, PAI, and ESR values. The other inflammatory markers (IL1, IL6, ferritin, TNF-α, fibrinogen) did not have a statistical significance. The significant direct correlation of pericardial effusion with CRP levels may suggest that pericardial inflammation, but not cytokine storm, may play an important part in the development of this complication in COVID-19 patients. Pericardial effusion can be also associated with clinical, biological, or electrocardiogram changes, especially when it is associated with pericarditis or myocarditis [9]. We noticed that there was a significant statistical correlation between pericardial effusion and chest pain as well as with the high median value of troponin and NT-proBNP.

The risk factors associated are difficult to evaluate, as there are many confounding comorbidities that can cause pericardial effusion, such as congestive heart failure, renal or hepatic failure, cancers, hypothyroidism, pulmonary embolism, malignancies, and pneumonia. However, it seems that patients with severe and critical forms, as well as those with higher age (>50 years) and children with multisystem inflammatory syndrome, are more likely to develop it, but large prospective studies to evaluate the true prevalence and significance of pericardial effusions in COVID-19 patients are lacking [4]. 

Furthermore, the actual incidence of pericarditis in cases of pericardial effusion remains uncertain because, at least early in the pandemic, healthcare workers had limited exposure to patients, leading to decreased utilization of echocardiograms and avoidance of aerosol-generating procedures like pericardial effusion drainage [8]. This is also a limitation of our study, as our cohort had limited access to electrocardiography and echocardiography. Another limitation was the retrospective single-center design and the relatively low number of patients with pericardial and pleural effusions.

## 5. Conclusions

Pericardial and pleural effusions can complicate COVID-19 infections. However, there are limited data in the literature about the prevalence or clinical importance of pericardial and pleural effusions in COVID-19 patients. In our study the prevalence of pericardial and pleural effusions in hospitalized patients was low, 5.3% and 8.3%, respectively, being associated with a number of clinical and biological parameters and some comorbidities. 

## Figures and Tables

**Table 1:** Baseline characteristics of COVID-19 patients with and without pericardial/pleural effusions.

Variables	All Patients (*n* = 337)	Group 1 (Pericardial Effusion = 18)	Group 2 (Pleural Effusion = 28)	Group 3 (without Pleural Effusion = 294)	*p*-Value
Age, years, mean (SD)	58.26 ± 14.58	66.89 ± 12.09	61.64 ± 17.03	57.52 ± 14.31	0.010 * 0.200 ** 0.015 ***
Sex %	Male—64.4% Female—35.6%	Male—66.66% Female—33.33%	Male—50% Female—50%	Male—65.3% Female—34.7%	0.836 * 0.097 ** 0.359 ***
BMI (mean)	29.31 ± 5.56	26.39 ± 3.14	29.11 ± 6.18	29.39 ± 5.51	0.024 * 0.889 ** 0.201 ***
Comorbidities	Hypertension—49%	With—77.8% Without—22.2%	With—71.4% Without—28.6%	With—45.6% Without—54.4%	0.012 * 0.013 ** 0.001 ***
	Diabetes mellitus—17.8%	With = 11.1% Without—88.9%	With—14.3% Without—85.7%	With—18.4% Without—81.65	0.445 * 0.611 ** 0.480 ***
	Chronic obstructive pulmonary disorder—2.1%	With—0.0% Without—100%	With—3.6% Without—96.4%	With—2% Without—98%	0.525 * 0.563 ** 0.903 ***
	Chronic renal disease—5.6%	With—17.9% Without—82.1%	With—17.9% Without—82.1%	With—3.4% Without—96.6%	0.021 * 0.001 ** 0.000 ***
	Chronic viral hepatitis—6.2%	With—3.6% Without—96.4%	With—3.6% Without—96.4%	With—3.6% Without—96.4%	0.004 * 0.543 ** 0.372 ***
Disease severity	Critical form—2.7%	With—11.1% Without—88.9%	With—7.1% Without—92.9%	With—1.7% Without—98.3%	0.022 * 0.125 ** 0.004 ***
	Severe form—58.8%	With—55.6% Without—44.4%	With—78.6% Without—21.4%	With—57.5% Without—42.5%	0.777 * 0.026 ** 0.215 ***
	Non-severe form—38.5%	With—27.8% Without—72.2%	With—17.9% Without—82.1%	With—23.3% Without—76.7%	0.321 * 0.007 ** 0.017 ***
Days from symptoms onset to hospital admission (median)	8 days ± 3.44	9.33 days ± 6.57	10.89 days ± 5.73	8.56 days ± 3.09	0.011 * 0.009 ** 0.103 ***

The *p*-value was represented separately for each group: * represents the *p*-value for the pericardial effusion group, ** for the pleural effusion group, and *** for the group without pericardial/pleural effusions.

**Table 2:** Clinical, biological and radiological characteristics of patients with and without pericardial and pleural effusion.

Variables	All Patients (*n* = 337), Median	Group 1 (Pericardial Effusion = 18), Median	Group 2 (Pleural Effusion = 28), Median	Group 3 (without Pleural Effusion = 294), Median	*p*-Value (Pericardial/Pleural Effusion vs. without Pericardial/Pleural Effusion
White Blood Cell count (cells/mm^3^)	6100.00 ± 3052.31	7499.44 ± 4408.81	7763.57 ± 4381.23	6651.56 ± 2786.48	0.138
Neutrophil count (cells/mm^3^)	4400.00 ± 2767.62	6016.17 ± 4300.58	6568.25 ± 4083.67	4962.43 ± 2453.67	0.038
Lymphocyte count (cells/mm^3^)	900 ± 532.02	936.67 ± 603.57	702.14 ± 281.36	1061.4 ± 536.37	0.005
Neu/Ly ratio	5.11 ± 5.43	9.15 ± 8.45	10.58 ± 8.90	5.96 ± 4.62	0.007
Platelets (cells/mm^3^)	197,400 ± 115,084.37	278,333 ± 339,422	228,303.57 ± 122,168.01	211,355.10 ± 83,003.96	0.261
LDH (U/L)	362.06 ± 239.05	349.56 ± 100.68	392.29 ± 133.02	360.39 ± 251.84	0.741
CRP (mg/L)	71.49 ± 64.99	101.82 ± 76.00	102.83 ± 80.07	67.70 ± 60.02	0.002
ESR (mm/h)	42 ± 23.56	54.77 ± 29.48	56.61 ± 26.76	42.66 ± 23.01	0.037
Fibrinogen (mg/dL)	507 ± 156.52	535.72 ± 144.31	578.98 ± 161.06	524.03 ± 157.05	0.203
IL-1 (pg/mL)	2.91 ± 143.77	22.90 ± 24.54	11.15 ± 19.69	28.83 ± 157.42	0.638
IL-6 (pg/mL)	101.80 ± 490	362.34 ± 469.04	269.16 ± 279.57	219.22 ± 508.46	0.413
PAI (ng/mL)	285.05 ± 318.36	391.24 ± 176.40	519.09 ± 676.67	338.88 ± 256.97	0.020
Serum ferritin (ng/mL)	602.20 ± 544.27	765.08 ± 550.53	915.17 ± 587.86	739.24 ± 541.70	0.345
TNF alfa (pg/mL)	11.10 ± 60.15	13.37 ± 9.63	12.46 ± 8.97	22.50 ± 64.51	0.393
NT-proBNP (ng/L)	30.00 ± 2437.06	2723.44 ± 5364.34	2441.59 ± 5994.08	292.22 ± 1575.53	0.000
Myoglobin (ng/mL)	138.83 ± 103.52	116.99 ± 49.92	164.26 ± 112.05	137.58 ± 104.69	0.573
Troponin (ng/mL)	0.04 ± 0.13	0.05 ± 0.07	0.12 ± 0.45	0.03 ± 0.04	0.287
D-dimers (x upper limit of normal)	2.41 ± 10.24	2.09 ± 2.58	2.90 ± 3.91	2.41 ± 10.89	0.997
HbA1c (%)	5.95% ± 1.18	5.92 ± 0.68	6.09 ± 1.06	6.30 ± 1.22	0.256
Heart rate (beats per minute)	89 ± 16.17	93.67 ± 13.59	92.61 ± 18.46	89.96 ± 16.03	0.239
Respiratory rate (per minute)	18 ± 3.4	19.06 ± 3.57	20.29 ± 4.54	19.15 ± 3.37	0.358
Saturation (%)	93% ± 11%	86% ± 21%	89% ± 54%	91% ± 10%	0.054
Pleuro-pericardial effusion (%)	12.8%	5.3%	8.3%		
Mediastinal lymph node images (%)	54%	61.1%	67.9%	52.7%	0.216
Respiratory failure (%)	61.7%	61.1%	85.7%	59.9%	0.067

The *p*-value represents the comparison between the pericardial and pleural effusions group and those without pericardial/pleural effusions. Abbreviations: LDH—lactate dehydrogenase; CK—creatine kinase; CK-MB—creatine kinase myocardial band; CRP—c reactive protein; ESR—erythrocyte sedimentation rate; IL-1—interleukin 1; IL-6—interleukin 6; PAI—plasminogen activator inhibitor; TNF alfa—tumor necrosis factor; HbA1c—glycated hemoglobin.

## Data Availability

The data presented in this study are available on request from the corresponding author.
